# Service dogs for autistic children and family system functioning: a constant comparative analysis

**DOI:** 10.3389/fpsyt.2023.1210095

**Published:** 2023-07-13

**Authors:** Sarah C. Leighton, Kerri E. Rodriguez, Leanne O. Nieforth, Marguerite E. O’Haire

**Affiliations:** ^1^OHAIRE Lab, College of Veterinary Medicine, University of Arizona, Tucson, AZ, United States; ^2^Human-Animal Bond Lab, College of Veterinary Medicine, University of Arizona, Tucson, AZ, United States

**Keywords:** assistance dogs, autism spectrum disorder, human-animal interaction, family systems approach, stigma

## Abstract

**Introduction:**

Service dog placements for autistic children are growing in popularity, yet findings to date are mixed. Moreover, no study to date has examined these placements through the lens of a recognized theoretical model. The purpose of this study is twofold: to explore experiences reported by caretakers of autistic children involved in a service dog program, and to contextualize findings within an established theoretical framework.

**Methods:**

A total of *n* = 50 caretakers of autistic children (*n* = 38 with and *n* = 12 without a service dog) were recruited through the national non-profit service dog provider Canine Companions. Participants completed an online survey through Qualtrics which asked open-ended questions about their experiences, both negative and positive.

**Results:**

Constant comparative analysis identified two high level themes, nested within a family systems approach framework: (1) Enhancing social functioning of the family system unit and (2) Fostering stability and strength within family system subunits. These themes interacted holistically to foster and reinforce family system resilience. Placements led to greater social inclusion for children and their families, acted as a highly individualized intervention, and decreased experiences of judgement and stigma. Perceived as members of the family, service dogs may coregulate with the autistic child and family members and can be a source of joyful connection within the family.

**Discussion:**

Results highlighted the service dog’s influence on the entire family (beyond the autistic child). Implications for service dog organizations suggest it may be helpful to account for family-wide impacts throughout the placement process. High standards on the part of provider organizations may minimize negatives, optimizing outcomes for both humans and canines. Ultimately, findings enrich our understanding of service dog interventions for autistic children.

## 1. Introduction

In the United States, about one in 54 children are diagnosed with autism spectrum disorder (1). Autism spectrum disorder is a neurological condition characterized by both strengths and challenges. The diagnostic criteria for autism have tended to focus more on challenges including social difficulties, restricted interests, and repetitive behaviors (2). However, while the presentation of autism is highly heterogenous and unique to each individual, research has also identified a deep sense of pride within the autistic and neurodiverse community and highlighted strengths such as adaptability, creative thinking, individuality, and visuospatial processing (3, 4). The prevalence of autism is higher in males than females, and has been on the rise in recent decades (1). This increase has largely been attributed to a combination of increased awareness, broadened diagnostic criteria, and increased identification within under-diagnosed populations, rather than an actual rise in incidence (5). In the United States, it is estimated that for each autistic child, families spend an additional $3,930–$5,621 annually in healthcare costs relative to children without autism (6). Moreover, neurodiverse individuals frequently experience stigma which can have an even greater negative impact on functioning than the characteristics of their mental condition itself (7). A broad range of established, emerging, and unestablished interventions exist for autism to optimize functional outcomes and adaptive skills (8). For example, for individuals under 22 years of age, established interventions (backed by thorough research demonstrating effectiveness) include story-based interventions and behavioral interventions. Emerging interventions (with some research demonstrating effectiveness) include music therapy, picture exchange communication systems, and exercise; and unestablished interventions (with no sound research demonstrating effectiveness) include animal-assisted therapy, movement-based interventions, and concept mapping (9). Autism interventions should aim to promote an increase in quality of life, empowerment, and fulfillment (10).

One such complementary intervention is placement with a service dog trained to support autistic individuals. Service dogs can be trained in any number of tasks depending on the needs of the individual. Tasks may include providing calming deep pressure, facilitating social interactions, retrieving dropped items, and participating in structured therapies (11). According to Assistance Dogs International, the accrediting body for assistance dog providers, service dogs for autism are currently the third most common type of assistance dog after guide dogs and mobility service dogs (12). A total of 64 accredited organizations train these types of service dogs, up from 19 in 2014 – a more than 3-fold increase in less than a decade (13). In contrast to other types of service dog partnerships, in which the service dog handler is also the individual receiving assistance, service dog placements for autism often involve three parties: the autistic child, the service dog, and a caretaker (or “facilitator”) who handles the service dog (11). The triadic (rather than dyadic) nature of these placements may lend additional complexity to the intervention; for example, the service dog’s primary bond may form with the caretaker rather than the autistic child (14), and it can be difficult for the service dog to accompany or support the child in school settings given that the handler would not be present (15).

To date, while some types of service dog placements can be considered an emerging intervention (e.g., service dogs for PTSD in the realm of mental health), service dog placements for autistic children remain an unestablished intervention due to the limited evidence currently available. While a handful of studies have examined the biopsychosocial outcomes of this intervention and revealed encouraging findings, results overall have been inconsistent and mixed, particularly when comparing studies with quantitative versus qualitative designs (16, 17). Based on the existing research, benefits appear to consistently include enhanced safety of the autistic child, the dog’s role as a social catalyst, improved emotional well-being, and enhanced self-regulation (16, 18–21). On the other hand, challenges can include increased burden in caring for the dog, canine behavioral issues, public access issues, and possible welfare concerns for the dog (14, 18, 22, 23). Perhaps unsurprisingly given the triadic nature of these placements, most studies identify impacts extending beyond the individual child to include the entire family, underscoring the importance of considering the family as a whole when evaluating outcomes (16, 22, 23). However, no study to date has attempted to examine this intervention in the context of an existing theoretical framework.

Thus, this study aimed to explore the experiences reported by caretakers of autistic children involved in a service dog program, including those with a service dog and those on a waitlist to receive a service dog. Using a qualitative approach, our goal was to contextualize findings within a broader established theoretical framework in order to develop a richer understanding of the service dog intervention for autistic children.

## 2. Materials and methods

This study reports a subset of qualitative findings from a larger mixed-methods, cross-sectional study. This study received ethical approval from the Purdue University Human Research Protection Program (IRB #1906022320). An exemption was obtained from the Purdue Institutional Animal Care and Use Committee (IACUC) since no interaction took place between the research team and any dogs.

### 2.1. Intervention

Service dog placements were made at no cost to the recipients by the non-profit organization Canine Companions, an Assistance Dogs International-accredited service dog provider in the United States and the largest service dog provider globally. Individuals with disabilities, or their caretakers, undergo a multi-step application process which includes submission of an application, reference forms from healthcare providers, a telephone interview, and an in-person interview (24). Eligible individuals are placed on a waitlist to receive a service dog. Canine Companions service dogs are Labrador Retrievers, Golden Retrievers, or Labrador/Golden Retriever Mixes purpose-bred for their role. Puppies are raised by volunteers for approximately 18 months before undergoing an additional 6–9 months of professional evaluation and instruction with Canine Companions training staff. Trained tasks include basic obedience (e.g., sit, down, loose leash walking), providing calming deep pressure, retrieving dropped items, and social greetings. As is common for this intervention, placements consist of a triad including the autistic child, the service dog, and a primary caretaker (often a parent) responsible for the service dog’s care and management. Placements occur onsite at training centers, with caretakers receiving 2 weeks of hands-on instruction in the service dog’s management, care, and safety. Canine Companions provides in-person and remote support throughout the duration of the placement.

### 2.2. Participants

A total of *n* = 50 caretakers (90% identifying as female) of autistic children were recruited through Canine Companions. Participants that completed the qualitative component of this study included *n* = 38 with a service dog and *n* = 12 without a service dog, but on the waitlist to receive one. All participants received unrestricted access to usual care. Among those with a service dog, time since placement ranged from 0.56–7.27 years (*M* = 3.75, *SD* = 1.96). Study eligibility criteria included (1) a community diagnosis of autism spectrum disorder, (2) the child being 5–17 years old, and (3) meeting provider eligibility criteria. Provider eligibility criteria included (1) child being at least 5 and caretaker at least 18 years old, (2) child having a disability, (3) needing a task (s) that a Canine Companions dog can provide, (4) child and caretaker cohabitating, and (5) caretaker being able to control, manage, and care for the dog.

### 2.3. Measures

Participants completed an online survey administered through Qualtrics experience management software (Qualtrics, Provo, UT) which included optional open-ended questions. Only the responses to open-ended questions are included in the present analysis. Participants waiting to receive a service dog were asked one open-ended question at the survey’s conclusion: “Is there anything else you would like to share about yourself, [child’s name], or your thoughts about a future [service dog]?” Participants with a service dog were asked six open-ended questions: (1) “What has [service dog’s name] been trained to do (i.e., a specific behavior, alert, or command) that has helped the most?” (2) “What autism spectrum disorder symptom has [service dog’s name] benefited or impacted the most with [child]?” (3) “How has [service dog’s name] positively impacted you as a caregiver?” (4) “How has [service dog’s name] positively impacted your family as a whole?” (5) “How has [service dog’s name] negatively impacted you, [child], or your family as a whole? (6) “Is there anything else you would like to share about [service dog’s name]?”

### 2.4. Analysis

We conducted a constant comparative analysis (25) to understand caretaker experiences and contextualize them within an established theoretical framework. Authors SL and LN read and re-read qualitative survey responses to identify similarities and differences and thereby develop initial categories (26). The team met regularly to discuss the analytic approach, align categories and codes, and identify areas requiring refinement. This iterative process continued until no new categories emerged [i.e., theoretical saturation, (27)]. Researchers then scanned literature containing potentially relevant theories and analyzed the fit of the coded data compared to theories. A single theoretical framework was identified (a family systems approach). This theoretical framework’s major tenets were evolved in further detail from an etic perspective, extracting exemplar quotes from participant responses (28). Member checks were conducted to assess whether findings conformed to the lived experiences of caretakers of children with service dogs for autism (26).

## 3. Results

Constant comparative analysis identified two primary themes, consistent with a family systems approach (29): Theme 1. Enhancing social functioning of the family system unit, and Theme 2. Fostering stability and strength within family system subunits. Taken together, these themes contribute to building the resilience of the family system as a whole.

### 3.1. Theme 1. Enhancing social functioning of a family system unit

Caretakers described four ways in which the service dog improved the family system’s functioning relative to other systems (i.e., on a macroscopic scale): As a social bridge, a social cue, a social buffer, and a social catalyst.

#### 3.1.1. Subtheme 1.1. The service dog as a social bridge

First, the service dog acted as a social bridge for both the autistic child and their family, inviting others to approach and interact. One caretaker shared, “she’s like a magnet, attracting all kinds of people over into our ‘Sphere.’” It gave caretakers “joy to see [the service dog] attract other children.” One participant shared an example of what this might look like in the context of school attendance:

When [child] was actively attending public school … I would sometimes take [service dog] to campus and friends who would not usually engage with [child] would approach us and ask about [service dog]. This was a social bridge for [child] and other students who knew [child] was the girl with the “sweet dog.”

The service dog’s role as a social bridge not only attracted others, but also encouraged proactive connection with others for some children. As one caretaker shared, “[child] has been able to approach children and start conversations more easily because he starts conversations off about his dog.”

#### 3.1.2. Subtheme 1.2. The service dog as a social cue

Beyond inviting attention, numerous participants shared that the service dog’s presence decreased stigma and judgement from others and increased their patience and tolerance. “People are more accepting of behaviors when he’s around,” shared one caretaker. Some autistic children use augmentative and alternative communication (AAC) devices to communicate, wherein a device (such as a tablet) can be used to generate speech. Added patience from community members was particularly helpful for these children:

It’s nice because the kids can pet the dog while [child] is answering questions. It takes a while to push the buttons to make a sentence. Kids wouldn’t wait before the dog. It’s almost like they want her to take her time so they can play with [service dog]. [Child] takes pride in it. We make all sorts of friends in line at Disneyland.

In some cases, the service dog’s presence served to make the “invisible” visible, in a positive way:

She is a quick indicator for people that there is a disability present, even if it’s just momentarily invisible while [child] is sitting quietly on a park bench, and allows them the time to adjust their behavior and expectations.

This lessening of stigma served to decrease isolation for the autistic child, ultimately improving their well-being: “[Service dog] has … helped [child] interact with his environment more and become less isolated. [Child] is a happier child and feels more accepted in the world with [service dog] in his life!”

#### 3.1.3. Subtheme 1.3. The service dog as a social buffer

Sometimes, the experience of interacting with the outside world was overwhelming for the autistic child. In these cases, the service dog provided a buffer and focal point, “directing [child]‘s attention to [service dog] instead of overwhelming situations or places.”

Sometimes, this buffering effect occurred passively through the dog’s mere presence. As one caretaker shared, “[child] finds comfort in putting her hand on [service dog] when out in public.” Another described that “having [service dog] always by his side directs [child]‘s attention to [service dog] instead of overwhelming situations or places.” Shared a third, “when you tell [child] about a hospital visit or a doctor appointment he is quiet for a minute then says can [service dog] come? And everything is better.”

In other cases, the buffering effect was accomplished through use of trained tasks. “Deep pressure [helps] with sensory overload. [Service dog] will hug [child] or sit on her feet or lean against her when waiting in line at a store or at school,” one participant shared. Another identified that “the commands that tell [service dog] where I need her to go are most helpful … These help me position her to best support [child] in specific situations or to be a ‘buffer’ between him and other people or activity.”

#### 3.1.4. Subtheme 1.4. The service dog as a social catalyst

For many families, the service dog acted as a social catalyst for community building. Above and beyond the dog’s role as a social bridge (inviting approach and interaction on an individual level), the service dog contributed to greater participation within the community on a systemic level: “[Service dog] has made the family feel and experience life as ‘normal’ might these last 3 years. [Service dog] is such a people-dog, she just brings the joy out of strangers – and that’s wonderful to be a part of.” Thanks to the service dog, families were able to “attend baseball games and other places that [child] very well may not have done.” By opening up the world for the autistic child, the service dog likewise opened up the world for the rest of the family:

Knowing that [child] has a friend [service dog] and that [service dog] helps [child] access public places makes it so that I can access public places. Making sure that [child] is part of the community with the help of [service dog] rather than isolated in our home means everything!

In some cases, the service dog did not merely connect or re-connect families to the community; they were themselves a source of new community. As described by one participant, the service dog “has helped me join the world of dog people; gives me something to talk to others about which is helpful given that it’s hard to engage socially.”

### 3.2. Theme 2. Fostering stability and strength within family system subunits

Within the family system itself, service dog placements fostered greater stability and strength in four key ways: As a member of the family themselves and a catalyst for improved family interactions, by coregulating with family members, as a highly individualized intervention for the autistic child, and as a source of joy. Additionally, we found that the service dog’s influence within the family was almost entirely positive for participants in this study, with a few specific exceptions. Finally, we identified that the service dog intervention’s influence extended even before placement for families on the waitlist.

#### 3.2.1. Subtheme 2.1. A catalyst for improved family interactions

Service dogs improved family system stability by smoothing interactions between family members. This influence occurred from within the family system: the service dog was, beyond a doubt, “not just a dog, [but] part of our family.” In several families, the service dog was perceived as equivalent to a sibling:

[Child] also sees [service dog] as a sort of brother, at times comparing herself to him in a mildly competitive sibling way—for instance, if [service dog] gets into poison ivy or mud in the back yard and gets “in trouble” for it, she will say ‘[Service dog], you are in trouble but I am not in trouble!

Family system structures shifted on a fundamental level to adjust for the addition of the service dog as a new family member. In many cases, this led to a direct transformation from imbalanced, unhealthy dyads between individuals (e.g., child-caretaker dyad, child-sibling dyad), to better-balanced triads with the addition of the service dog.

Perhaps most critically, the service dog enabled a diffusion of tension and restoration of healthier equilibrium in the relationship between child and caretaker. In many families, caretakers described sacrificing their own sleep or needs in their efforts to care for and meet the child’s needs. The development of a caretaker-child-service dog triad is inherent to the structure of these placements, and this created space for the caretaker’s needs to be met as well. “I feel like we are a team,” shared one participant, “[service dog]‘s always got my back and is there to help in any way she can.” Another described that “because [child] has a best friend at home that she is engaged with, I have time to do household chores.” A third shared, “[service dog] gives [child] a break which gives us a break.” For several participants, this shift took place most notably in the domain of sleep:

[Child] would not sleep. I would have to lay on her, practically on top of her to give her deep pressure. One night I thought a dog could do this and I could do the dishes and finish laundry instead of laying here and falling asleep before she did. [Service dog]‘s main job was to get my girl to sleep … sleep is not our problem anymore.

Having him with her at night meant I started sleeping through the night. I was up a couple of times a night with her before he came home with us and I cannot express how good it felt to start sleeping well.

The child-sibling dyad was also improved with the welcoming of a service dog to the family, creating “something positive and wonderful to bond over”:

He is also a significant support to our entire family and allows our three children to bond in ways they couldn’t before we had [service dog]. They have their love for him in common, and this helps them connect. My other two have resentment towards [child] for the pain they feel he has caused them over the years.

The joy I feel when I see the kids bonding over [service dog] remains even now that they are all essentially teenagers – they still interact with and chat about [service dog] pretty much daily, and this always brings a smile and a sense of relief and even hope for them staying connected as siblings in spite of our challenges.

Overall, for participants in this study, the service dog was a positive presence who drew focus away from challenges or negatives; loving the service dog was something the entire family had in common. “He brings us together as a family, because we all love him so much,” shared one participant. Another described that “everyone loves to see [service dog] make [child] happy.” The everyday routine surrounding the service dog supported a healthier family dynamic:

Walking a dog, grooming her, playing fetch with her, dressing her up or just snuggling with her might not seem like a big deal, but all of it has had a strong impact on our family as a community in relationship with each other.

#### 3.2.2. Subtheme 2.2. Coregulating with individuals to foster homeostasis

While relationships between family members partly influence family system stability, the well-being of individual family members is equally important. The service dog further increased stability by improving the well-being of individual members of the family through coregulation, or the development of a shared emotional system (30). Through this coregulation, the service dog fostered physiological equilibrium (homeostasis) thanks to their trained tasks, presence, calm demeanor, and intuition. Described one participant, “if any one of us is upset, [service dog] walks over and lays his head on our lap.”

For the autistic child, the trained task of deep calming pressure was mentioned by far the most. “[Service dog] is trained to cover or visit to give deep pressure,” explained one caretaker. “When [child] needs input or is upset he likes to have [service dog] lay or sit partially on him.” In doing so, the autistic child was able to achieve “calm and peace,” which for many caretakers “immediately reduced my stress levels enormously.” Even beyond trained tasks, the service dog – as a source of calm themselves – was “generally a calming, co-regulating influence.” One participant shared, “[child] calls him her patronus (Harry Potter reference) because every time she touches him he gives her joy. He is like an island of calm she can reach out to any time.” Caretakers repeatedly described the role of the service dog’s intuition in this relationship:

The bond between [child and service dog] is amazing and it’s so impressive to see how [service dog] knows when [child] needs him and he has to do his job. [Service dog] doesn’t even need a command to help [child]. He will hear [child] crying or upset and he comes running to help. Our dog is amazing!!

Notably, although it was not a trained task for service dogs from this organization, multiple participants positively and specifically described the service dog licking away tears:

[Service dog] is priceless in the hospital. A few times [child] hallucinated and he was the only one that could calm her down … When she is upset she runs to him, buries her head in his fur and cries and tells him how she is feeling … If she cries, he licks her tears off, which helps her a lot.

The service dog also coregulated and demonstrated emotional intuition with caretakers. As one participant shared, “[service dog] and I have a very, very special connection. He knows that he is [child]‘s boy—but knows that I have his back, that I am in charge and that I will take care of all of us. [Service dog] has a tremendous calming influence on all of us.” Another caretaker described that “he picks up on my stress. When I start to get upset he will come up and lick me or run and grab a toy from his basket to bring to me.” This bond was motivating and rewarding to caretakers:

He is love! He loves to cuddle and picks up on my emotions. He is motivation for getting out for walks because he is depending on me. He makes me feel good and accepted and important. When it’s a bad day, he is always there for being cuddled and doesn’t demand anything of me.

The positive impacts of the service dog’s presence extended to the entire family. One caretaker described that the service dog “can sense when any of the children are having a bad day and she will love on them.” Another shared that “the whole family just loves her. My younger daughter is less anxious, my husband loves to snuggle with her while we watch tv. She cheers up my mother-in-law when we visit her.”

Finally, in three cases, the primary bond did not develop between the service dog and the child, but rather the service dog and a parent. Interestingly, in all three of these cases, the service dog took on a working role with that parent instead:

My wife, who has a chronic health condition, spends the most time with [service dog] by far. … We both agree, as a “service dog,” [service dog] is working her magic almost purely with [my wife] … who actually needs companionship and moral support. So, she’s doing her job, but she’s doing it in a way we hadn’t originally planned.

[Dad] is [service dog’s] main handler and spends the most time with [service dog] when [child is] at school. He is retired due to disability. [Service dog] has helped him so much.

#### 3.2.3. Subtheme 2.3. A highly individualized intervention

For most of the autistic children in this study, placement with a service dog was a highly individualized intervention that helped in targeted ways beyond coregulation, ultimately enhancing independence.

For some, the service dog gave the child a “sense of purpose and identity,” teaching the child about “independence by helping with chores and feeding,” increasing the child’s “confidence and self-worth,” and acting as a “motivating factor” to accomplish other tasks. For many children, having the opportunity to give the dog a command (i.e., cue to perform a task) was motivating: “[Child] will do almost anything just to be able to give [service dog] a command.” For other children, benefits were seen in physical health: one caretaker shared that their child’s “irregular gait and walking tempo has improved TREMENDOUSLY by walking with [service dog] on harness regularly.” One child experienced improvement to sensory sensitivities “through touching [the service dog] and [the service dog’s] care.”

In several cases, benefits surpassed the service dog’s original training. One caretaker shared, “[service dog] alerts me when [child] has a seizure – he is not trained for it – he just started doing it, but it is priceless and lets me grab her and set her down saving her injury.” Another described the service dog helping the child fall back asleep at night, saying “I know he’s not trained for that, but before we had him, she woke up often in the night. Now if she starts to stir, he cuddles up to her and often she goes back to sleep.”

Above and beyond trained tasks, the service dog was also a “best buddy” to the autistic child, providing “unconditional love and friendship” where, in some cases, the child may have been isolated. As one participant shared, “[child] does not have the kinds of friends he can rely on, or hang out with outside of school. [Service dog] is his best friend and he considers her part of his family.” Another described that for their child, the service dog “is her rock. When she is overwhelmed, she hides with him, he lays on her and she breathes. When she is in pain, he is the one who can help calm her down.” For many children, the service dog was incorporated into every-day activities and conversations, just as a human friend would be:

[Service dog] is [child]‘s best friend. She talks with him ALL the time (even right now as I type). I mean ALL the time, whether he is with us or not. She explains about what it is like to be a human to him, tells him about social etiquette, tells him what different words mean, what are kind/mean and safe/unsafe behaviors, tells him about things she has experienced … she has a best friend who is very happy to hear all that she has to say.

[Child] looks at [service dog] as his best friend. He wants to show her everything he does. He just wants to see her and know she is there for everything he does from getting dressed to making his bed.

They sleep with either her legs over him or his arms around her shoulders or holding hands (it is adorable).

#### 3.2.4. Subtheme 2.4. A source of joy, laughter, play, and calm

The service dog’s presence within the household was a source of joy, laughter, play, and calm for families. Caretakers described positive changes in “the overall mood” of the family. As one participant explained, “she brings laughter, joy, playfulness, and a motivation to be active to each of us.”

This joy stabilized and strengthened bonds between family members: “[Child] and I also laugh a lot about his antics—so he brings a lot of laughter into our home,” one participant shared. Another described that “the whole family is happier having [service dog] around, even though he’s [child]‘s dog, his training makes him more sensible and he comforts and plays with everybody.”

#### 3.2.5. Subtheme 2.5. An almost entirely positive influence

While the service dog intervention can promote stability, it may also lead to added challenges. However, when asked about negatives, by far the most common response was that “there has been nothing negative” about having the service dog. “I honestly cannot think of one negative impact [service dog] has had on our family,” shared a caretaker. One participant elaborated that they “expected an adjustment or change in routine when we graduated and it never happened. Things only got easier for us.”

Of the challenges that some participants shared, a few consistent areas were mentioned. Most common was the volume of shedding: “The amount of HAIR [service dog] produces is incredible … really, I cannot believe it.” “Really and truly, the only downside to [service dog] … is ALL THE DOG HAIR! If she did not shed so much, she would be pretty darn close to perfect.” More broadly, for a minority of caretakers, the added responsibility could be a burden; as one caretaker described, “sometimes [service dog] feels like an added responsibility when things are hard around her (like if [child] has a seizure and needs medical attention).”

While no participants in this study described active financial hardship relating to the service dog, for some, the potential of future costs were a salient concern. One participant shared that as the service dog “is getting older, vet expenses are increasing a bit. Hopefully that will not become a major issue.” In some families, this was an ongoing source of worry:

I worry sometimes about the cost of medical care, should [service dog] fall ill. Of course we’d do anything for him, but I’ve heard stories from friends about the thousands they’ve had to pay for pet surgeries etc. Money isn’t easy for us – [child]‘s interventional treatments from age 5–9 were not covered by insurance.

Although for most participants the service dog partnership came with none, few, or only minor challenges, a sole participant shared that “the negative things about [service dog], have been bad enough at times, I just wish we had not gotten him. Thank goodness they are not thoughts I have often, but they are there.”

Notably, the service dog’s high degree of training and preparation for the role appeared key to the lack of negatives:

[Service dog] is just such a love, she provides all the benefits of a regular dog but without any hyperness, overexcitement, barking, yipping, nudging, or all the things regular dogs do that could make that aversive to a guy like [child].

[Service dog] is the dog love of my life, and I loved dogs before this, including doing years of rescue work. But despite all my knowledge, I could not have trained her this well. I think for me she provides all the benefits of a regular dog, plus without all the things that I could find aversive too.

The service dog’s level of training was not only crucial to the lack of downsides, for some families, it was essential to their ability to achieve service dog partnership in the first place. As one caretaker shared, “we would not have been able to have a poorly trained dog in our home because it would scare [child].” Another described that “when we got [service dog] there was no way that we could have gotten a pet dog because I could never have trained it.” The importance of the dog’s training and suitability for the working role was not only important for the family, it was also important to the dog’s welfare: “I think that [service dog] is trained to be with us, that we do not have to worry about him reacting to us in a bad way. He is always happy to see us and is gentle and calm and tolerates a lot of loud noises and sudden movements that might scare another dog.”

#### 3.2.6. Subtheme 2.6. Influence of the intervention for families on the waitlist

For families on the waitlist to receive a service dog, the intervention had an influence even prior to partnership by providing hope and excitement “at the prospect of what [a service dog] can offer.” As one family shared, “the thought of receiving a [service dog] has given us such hope for the future.” Another caretaker identified specific goals: “We hope that [child] really thrives in caring for his dog and taking responsibility to walk him every day. His goal is to master all the commands so the dog becomes his best friend.” The decision to apply for a service dog was one founded in optimism and excitement for the future:

We are a dog-loving family and have been on the lookout for getting an appropriate dog which will bring joy to the family and above all help [child] with the various social and emotional issues that she has. We are very excited that we have gotten the opportunity to get a [service] dog and strongly believe that it would change [child]‘s life and ours too.”

## 4. Discussion

The purpose of this qualitative study was to explore the experiences reported by caretakers of autistic children involved in a service dog program, and to contextualize results within a broader established theoretical framework. Through a constant comparative analysis, we identified that the experiences reported by caretakers were best explained through the framework of a family systems approach (29). Analyses revealed two primary themes. Theme 1, enhancing social functioning of the family system unit, included subthemes of the service dog as a social bridge, social cue, social buffer, and social catalyst. Theme 2, building strength and stability within family system subunits, included 6 subthemes: A catalyst for improved family interactions; coregulating with individuals to foster homeostasis; a highly individualized intervention; a source of joy, laughter, play, and calm; an almost entirely positive influence; and the influence of the intervention for families on the waitlist.

Previous literature has called for autism and disability research to take a family systems approach, recognizing that the well-being of an individual family member cannot and should not be fully disentangled from that of the family system [e.g., (29, 31)]. Family systems approaches incorporate family systems theory (32) to understand interfamilial processes and extend beyond these to understand the processes through which the family system interacts with external systems (communities, schools, other families, etc.) (29). The family systems approach conceptualizes these as microscopic and macroscopic lenses, respectively; from an ecological systems standpoint, the microscopic and macroscopic lenses can be considered to correspond to the micro-and meso-levels of a family ecosystem (33). Familial resilience is a key component of a family systems approach, impacting the family’s ability to respond to challenges, balance the needs of individuals, maintain interfamilial bonds, and engage with their community. Our study extends family systems approaches (including familial resilience) to a new context: a service dog intervention for families with autistic children.

Results found that the service dog has an impact on the entire family (beyond the autistic child). This finding is well aligned with results previously reported in autism service dog literature (16, 34) and assistance dog literature more broadly [e.g., (35–37)]. Research has identified that these placements can strengthen interfamilial bonds, impact wellbeing of individual family members, facilitate resilience processes, and increase social participation for the entire family; however, they can also lead to new challenges. This highlights an important consideration for service dog provider organizations, which may focus primarily on the individuals involved in the triadic placement (the caretaker, who cares for and handles the service dog, and the autistic child). However, family unit makeup can vary widely, from single-parent-single-child families to many other forms and sizes of family units. For these families, interventions focused solely on the child and/or the parent are unlikely to be fully effective (38). Accordingly, provider organizations should recognize family-wide impacts and that familial resilience processes (i.e., the ability to balance stressors and marshal resources) can influence the family’s ability to engage in an intervention at each step and thereby impact outcomes (39). Specifically, provider organizations should identify family unit makeup as part of the application process and incorporate the entire family into the intervention by setting expectations and accounting for each family member’s needs within each treatment component.

### 4.1. Theme 1. Enhancing social functioning of the family system unit

The first theme, enhancing social functioning of the family systems unit, takes a macroscopic view to understand the service dog’s influence on the family’s interactions with other systems such as their community and social groups. We found that the service dog may enhance and even increase these interactions. This finding is particularly salient given research that families of autistic children may experience social isolation, driven by difficulties participating in social activities and a lack of understanding from members of the community with regard to behaviors common for autistic children (31). Social support is a known moderator of negative outcomes (including depression, social isolation, and relationship difficulties), wherein decreased social support can increase the negative impacts of parental stressors for families of autistic children (40). Notably, we also found that families with a service dog perceived decreased social stigma and judgement from community members, in line with prior findings in autism service dog literature (34, 36). Experiences of social stigma and social acceptance appear to vary based on the type of service dog interventions. For example, social acceptance and recognition are among the most common benefits reported by individuals with hearing dogs (41), but stigma and judgement are among the biggest *negatives* reported by veterans with service dogs for PTSD (37, 42). Although no participants in our study experienced this as a negative, one study has previously identified that the experience of increased public visibility may be unwanted for some caretakers (20). Given these differential findings, an interesting area of future research would be to examine the social stigma and discrimination experiences of handlers across different types of disabilities and service dog placements.

While service dogs for autism have frequently been discussed in the context of school [specifically, legal challenges and confusion in this context; e.g., (15, 43)], this topic was absent from our findings due to this population not being encouraged to engage in this practice. Given that this will likely continue to be an area of discussion given legal and logistical complexities, further research with more targeted questions is warranted to better understand the experiences of handlers of service dogs for autism with regard to school.

### 4.2. Theme 2. Building strength and stability within family system subunits

The second theme, building strength and stability within family systems subunits, takes a microscopic view to understand the impacts on and between individuals and family subunits. At a high level, the service dog was clearly identified as an individual family member themselves rather than a separate entity. Conceptualizing animals as individuals within a family unit aligns with a biocentric orientation that recognizes and respects the deep connections humans can form with other species (44). Through their position within the family system, we found that the service dog may contribute to strengthening and stabilization. In other words, the service dog may foster increased family resilience internally. Indeed, prior research examining pet dogs in a therapeutic context has identified that because of this familial integration, pets – and in this case, service dogs – can be important components of the family’s healing team, strengthening family resilience (45).

Research suggests that an autism diagnosis can assist in developing resilience for families and that involvement of the entire family in interventions can lead to greater positive outcomes [e.g., (39, 46)]. Specific pathways for developing family resilience may include establishing routines, family time and togetherness, and social support (29, 47). These pathways map well onto a service dog intervention: routine can be created through the dog’s day-to-day needs, and for some children, taking responsibility for the dog’s care was a major benefit; the service dog was a source of joy, laughter, and play, thereby promoting togetherness and family time around the dog and dog-related activities. Finally, social support was improved through the dog’s role as a social bridge and catalyst. This last element of social support speaks to the holistic interaction between Theme 1 and Theme 2, and further reinforces our recommendation that service dog providers account for the entire family unit throughout the service dog placement and ongoing support process. Similar recommendations have been made in the context of service dogs for veterans with PTSD, and it would be reasonable to consider that this may be a best practice for service dog interventions of any type (37).

When family systems theory was first developed, it was proposed that triads can be considered the fundamental family building block and that the addition of a third individual can help ease tensions within unbalanced dyads (32). This has interesting applications in the context of a triadic service dog intervention; this rebalancing process aligns well with observations from participants in this study who experienced a diffusion of tension between child and caretaker or child and siblings with the addition of the service dog. Moreover, the service dog appeared to coregulate with family members individually, acting as a homeostatic regulator helping achieve physiological equilibrium – a known phenomenon which has been described in pet dog literature more broadly (45). This occurred not only through the service dog’s trained tasks (for the autistic child), but also through their presence and bond, echoing findings in service dog literature more broadly that speak to the importance of not only trained, but also untrained, behaviors (48).

In a few notable cases, service dogs in this study bonded not with the autistic child but with a parent. While prior literature has mentioned difficulties in child-dog bonding [e.g., (23)], these situations have previously been characterized as resulting from elements of the child’s disability, such as motor control or communication difficulties. These stand in contrast to the current study; in each case the dog appeared to have developed a working relationship with a different family member. It’s possible that the development of such a relationship directly interfered with bonding with the autistic child, but equally possible that in the absence of a strong bond forming between the service dog and the autistic child (for any number of reasons), the dog naturally gravitated towards another family member. An important line of future research will be to identify any factors – human or canine – that may be predictive of successful bonding between service dog and child, or whether there are cases where it is in fact *more* helpful for the service dog to form a primary bond with the caretaker instead. Some initial work has begun to characterize first interactions (49), and similar methods could be employed in a longitudinal design to begin identifying associations between early interactions and future bond strength. Ultimately, these findings could provide critical insights for service dog providers and health care practitioners to improve recommendations as to whether a service dog intervention would be appropriate, and if so, how to maximize efficacy.

It was apparent that placement with a service dog is a highly individualized intervention for autistic children. Given that the presentation of autism can itself be highly variable and unique to each individual, this is not surprising; however, it may shed some light on the disparities between qualitative and quantitative research findings on this topic (17). For example, if improvements are highly variable from domain to domain, standardized quantitative measures may result in null findings within a larger group. To account for this, future quantitative research should be thoughtful about measure selection and analytic strategy. Interestingly, when considering variation within the intervention, prior research on service dogs for autism frequently identify tethering (i.e., physically linking the autistic child to the service dog to prevent bolting or running away) as an important and necessary part of the intervention [e.g., (18)]. However, other research – including the present study – has identified benefits even in the absence of this task, which not all service dog providers train [e.g., (20)]. It is possible that individuals self-select when identifying a service dog provider based on their needs and the trained tasks offered, further underscoring the variable and individualized nature of the intervention.

Challenges raised in the current study included the service dog’s shedding, the added burden of service dog-related responsibilities, and stress about potential future veterinary expenses. However, the notable lack of negatives reported by participants in this study appears to stand in contrast to prior research on service dogs for autism. Studies have highlighted issues including ongoing training challenges, public access issues, added burden of care, financial impacts, and difficulty bonding as negatives of service dog placements for autistic children (20, 34). This discrepancy may be due to the way that this study asked caretakers about drawbacks (“How has [service dog’s name] negatively impacted you, [child], or your family as a whole”). Other studies have used open-ended text boxes to ask about “constraints of having a service dog” (22) or used semi-structured interviews to probe negative experiences in detail (18, 20, 23). Differences in experiences, including drawbacks, could also be due to differences across service dog providers. Adherence to high standards from service dog providers, including participation in accreditation processes, is important to minimize challenges experienced by autistic children, their families, and service dogs – ultimately optimizing outcomes and setting humans and canines up for success.

### 4.3. Limitations

Several limitations should be considered when interpreting these findings. This was a cross-sectional study, whereas both family systems and the needs of autistic children are known to evolve over time; thus, family functioning likely also evolves over time. Participants in this study were recruited from a single, United States-based service dog provider. While this increased standardization and homogeny of the intervention within this study, results may or may not be applicable to individuals participating in programs from other providers or in other countries. Additionally, surveys were completed by caretakers, and therefore responses reflect their personal experiences. It is possible that the autistic child or other family members would have shared different opinions or experiences if they had been the individual answering the questions. This study also did not examine the welfare of the service dogs themselves. Future studies should endeavor to include both human and canine outcomes, especially in light of findings that there could be welfare concerns in some cases (14). Finally, given that participants undergo a multi-step application process and 2 week, onsite training program as part of receiving the service dog, it is also possible that survey responses were influenced by the instruction and expectations set through interaction with the provider organization, and by the quality of the relationship between the two parties. In turn, it is likely that the provider’s language and content are influenced by reports from past clientele.

### 4.4. Conclusion

This qualitative study of service dog placements for autistic children lends insight into the experiences of caretakers, children, and families involved in a service dog intervention. Overall, service dog placements appear to impact and foster resilience within the entire family (beyond the autistic child) and were best understood through the lens of a family systems approach framework. Placements led to greater social inclusion for families, acted as a highly individualized intervention for the autistic child, and decreased experiences of judgement and stigma. Perceived as members of the family, service dogs may coregulate with individual family members and can be a source of joy and positive connection within the family. The two themes (1. Enhancing social functioning of the family system unit and 2. Building strength and stability within family system subunits) interact holistically in that the family’s resilience is strengthened through increased social support, fostering of homeostasis on an individual level, and increasing internal family stability. Implications for service dog organizations suggest it may be helpful to account for family-wide impacts throughout the placement process. High standards on the part of provider organizations may minimize negatives for children and their families, optimizing outcomes for both humans and canines. Overall, this study enriches and expands our understanding by extending a family systems approach in a novel context: that of a service dog intervention for families of autistic children.

## Data availability statement

The original contributions presented in the study are included in the article/supplementary material, further inquiries can be directed to the corresponding authors.

## Ethics statement

The studies involving human participants were reviewed and approved by Purdue University Human Research Protection Program (IRB #1906022320). The patients/participants provided their written informed consent to participate in this study. Ethical review and approval was not required for the animal study because an exemption was obtained from the Purdue Institutional Animal Care and Use Committee (IACUC) since no interaction took place between the research team and any dogs.

## Author contributions

KR and MO contributed to the conception and design of the study. SL and LN conducted the data collation and analysis. SL wrote the first draft of the manuscript and SL, KR, LN, and MO contributed to the manuscript revision. All authors contributed to the article and approved the submitted version.

## Funding

This project was supported in part by the Human Animal Bond Research Institute (#HAB19-011), Nestlé Purina, and Clifford B. Kinley Trust. This publication was made possible with support from the Indiana Clinical and Translational Sciences Institute which is funded in part by Award Number UL1TR002529 from the National Institutes of Health, National Center for Advancing Translational Sciences, Clinical and Translational Sciences Award (KR). The content is solely the responsibility of the authors and does not necessarily represent the official views of funders.

## Conflict of interest

The authors declare that the research was conducted in the absence of any commercial or financial relationships that could be construed as a potential conflict of interest.

## Publisher’s note

All claims expressed in this article are solely those of the authors and do not necessarily represent those of their affiliated organizations, or those of the publisher, the editors and the reviewers. Any product that may be evaluated in this article, or claim that may be made by its manufacturer, is not guaranteed or endorsed by the publisher.
